# Photoluminescence of Argan-Waste-Derived Carbon Nanodots Embedded in Polymer Matrices

**DOI:** 10.3390/nano14010083

**Published:** 2023-12-27

**Authors:** Corneliu S. Stan, Noumane Elouakassi, Cristina Albu, Ania O. Conchi, Adina Coroaba, Laura E. Ursu, Marcel Popa, Hamid Kaddami, Abdemaji Almaggoussi

**Affiliations:** 1 Faculty of Chemical Engineering and Environmental Protection, Gh. Asachi Technical University of Iasi, D. Mangeron 73 Ave., 700050 Iasi, Romania; cristina.albu@tuiasi.ro; 2 Innovative Materials for Energy and Sustainable Development (IMED-Lab), Faculty of Science and Technology, Cadi Ayyad University, Av. Abdelkrim Khattabi, B.P. 511, Marrakech 40000, Morocco; noumanelouaskassi0641@gmail.com (N.E.); h.kaddami@uca.ma (H.K.); a.almaggoussi@uca.ma (A.A.); 3 Conditions Extremes Matériaux Haute Temperature et Irradiation (CEMHTI), UPR 3079, CNRS, Université d’Orléans, 45100 Orleans, France; conchi.ania@cnrs-orleans.fr; 4 Centre of Advanced Research in Bionanoconjugates and Biopolymers, “Petru Poni” Institute of Macromolecular Chemistry, Grigore Ghica Voda 41A Alley, 700487 Iasi, Romania; adina.coroaba@icmpp.ro (A.C.); ursu.laura@icmpp.ro (L.E.U.); 5 Academy of Romanian Scientists, Ilfov Street, 050054 Bucharest, Romania; 6 Sustainable Materials Research Center (SusMat-RC), Lot 660-Hay Moulay Rachid, Ben Guerir 43150, Morocco; 7 Applied Chemistry and Engineering Research Centre of Excellence (ACER CoE), Advanced Organic Optoelectronic Laboratory, Mohammed VI Polytechnic University (UM6P), Lot 660-Hay Moulay Rachid, Ben Guerir 43150, Morocco

**Keywords:** carbon dots, polymer nanocomposites, photonic conversion materials

## Abstract

In this work, photoluminescent (PL) carbon nano dots (CNDs) prepared from argan waste were embedded in highly optical transparent poly(styrene-co-acrylonitrile) (PSA) and cyclo-olefin copolymer (COC) matrices, which were further processed into thin films. In the first step, the luminescent CNDs were prepared through thermal processing of fine-groundargan waste, followed, in the second step, by direct dispersion in the polymer solutions, obtained by solving PSA and COC in selected solvents. These two polymer matrices were selected due to their high optical transparency, resilience to various environmental factors, and ability to be processed as quality thin films. The structural configuration of the CNDs was investigated through EDX, XPS, and FTIR, while DLS, HR-SEM, and STEM were used for their morphology investigation. The luminescence of the prepared CNDs and resulted polymer nanocomposites was thoroughly investigated through steady-state, absolute PLQY, and lifetime fluorescence. The quality of the resulted CND–polymer nanocomposite thin films was evaluated through AFM. The prepared highly luminescent thin films with a PL conversion efficiency of 30% are intended to be applied as outer photonic conversion layers on solar PV cells for increasing their conversion efficiency through valorization of the UV component of the solar radiation.

## 1. Introduction

Photoluminescent carbon nano dots (CNDs) prepared from natural sources [1] could be a convenient approach due to the abundance of the raw materials from various vegetal and agricultural wastes available in large quantities. Besides the valorization of these readily available resources, the resultant CNDs are biocompatible [2] in most cases, and, according to the chemical composition of the precursors, the application range could vary from optoelectronic [3,4] and sensing [5,6] devices to biomedicine, including anti-tumoral activity [7,8,9] or medical imaging [10] such as MRI investigation techniques [11]. Typically, the CND structure consists of a surface-rich functional group-decorated graphitic core with a disordered configuration containing various atomic species (i.e., N and O) intercalated between the sp^2^ carbons [12,13].

One of their most interesting features is their photoluminescence, with a particular excitation-dependent emission [14,15] which is rather unique among fluorescent materials. The mechanism of the radiative processes involved in photoluminescent (PL) emission is still unclear but later studies highlight the importance of the surface functional groups and defects located within the graphitic core [16,17]. The efficiency of the radiative processes ranges from a few percent to an impressive 90% quantum yield [18] depending on the precursors, preparation approach, and certain strategies of surface chemistry modifications or doping of the graphitic core with various atomic species [19,20]. In most cases, the reported emission is located within the blue region of the visible spectrum, but green [21,22], red [23], and NIR [24] emission CNDs have been reported. In this regard, it has been reported that characteristics of the emission are markedly dependent on the dispersion medium [25].

CNDs are typically prepared following physical and chemical approaches [26]. The last ones (e.g., hydrothermal [27], pyrolytic [28], and thermal [29] processing) are usually the preferred strategic route due to their simplicity and the variety of the resulting CNDs in terms of composition (e.g., surface groups and graphitic core) adapted for an intended application. Besides the synthesis strategies, another important aspect in the preparation of CNDs is the choice of the precursor, and the use of abundant natural sources is most appealing [30,31]. In this sense, a wide variety of carbon-rich natural sources has been explored as precursors, covering a diversity of wastes such as plant wastes from agricultural and food processing [32], dried leaves [33], palm kernel [34], collagen [35], algae [36], or orange waste peels [37], among the many reported works with notable results.

Regarding applications and especially in those targeting optoelectronic devices, CNDs are hardly to be used directly, as resulted from synthesis. CND dispersions tend to gradually lose their PL emission intensity, due to a marked agglomeration of the nanosized particles into large clusters. This process depends on the dispersion solvent used, with polar solvents (i.e., water and EtOH) favoring the clustering and thus rendering a fast decay of the PL (within few days) [38]. Hence, for the applications where the long-term stability of the PL emission is required, the encapsulation of the CNDs in a suitable matrix appears an interesting approach to preserve their structural integrity and PL properties [39]. For this, the compatibility between the CNDs and the embedment medium is a critical requirement. Another important aspect is the transparency within excitation/emission wavelengths range. In this regard, optically transparent polymer matrices can provide both the long-term preservation of CNDs structural and PL emission stability. This is most outstanding for their use as photonic conversion layers in photovoltaic applications. As a few examples, CNDs have been embedded in poly(methylmethacrylate) [40], poly(vinyl-alcohol) [41], poly(dimethylsiloxane) [42], polystyrene [43], poly(vinylchloride), or polycarbonate [25].

In this work, CNDs with remarkable PL emission have been prepared through thermal processing of argan wastes, and they have been dispersed in poly(styrene-co-acrylonitrile) (PSA) and cyclo-olefin copolymer (COC) solutions and further processed in thin layers. The argan waste was selected as a precursor due to its availability as a low-cost material resulting from the argan oil production [44] through cold pressing of the argan kernels. Indeed, the production of argan oil represents an important socioecoenomic resource for Morocco, with over 3000 tons of oil produced per year, representing a percentage of argan cake wastes generated. On the other hand, the polymer matrices were selected due to their high optical transparency, resilience to various environmental factors, and ability to yield high quality thin films. Our data have shown that, through the embedment of the CNDs in those polymer matrices, the quantum efficiency of their photoluminescent emission is markedly increased. The prepared CND–COC/PSA nanocomposites were tested with notable results as thin photonic down-conversion layers aiming to improve the overall efficiency of the conventional photovoltaic (PV) solar cells by taking advantage of the UV radiation contained in the solar spectrum (~8% at the earth level).

## 2. Materials and Methods

### 2.1. Materials

Argan cake waste obtained during argan oil preparation through cold pressing (provided by a local Moroccan co-operative) was used as precursor of the CNDs. Ultra-pure distilled water (Millipore-Direct Q, Millipore, San Salvador, Salvador), ethanol (EtOH), chloroform (CLF), and tetrahydrofuran (THF) (Merck-Sigma-Aldrich, Bucharest, Romania) were used as dispersion media and solvents of the polymer matrices. Poly(styrene-co-acrylonitrile) (PSA) (Mw = 165,000) and cyclo-olefin copolymer (COC) pellets (Mw = 180,000) were also supplied by Merck-Sigma-Aldrich, Bucharest, Romania.

### 2.2. Methods

The infrared (FT-IR) spectra were recorded in the 400–4000 cm^−1^ range using a Shimadzu IR Affinity 1S spectrometer (Shimadzu Corp., Kyoto, Japan) according to the KBr method. The EDX investigation was performed on a Verios G4 UC Scanning Electron Microscope equipped with an EDS, EDAX Octane Elite energy-dispersive spectrometer (Thermo Fisher Scientific, Waltham, MA, USA). XPS analysis was performed on a K-Alpha Thermo Scientific spectrometer using Al-Kα (1486.6 eV) radiation. Processing of the XPS spectra was performed using the Avantage software (ver. 5.9922), with energy values referenced against the C1s peak of adventitious carbon at 284.6 eV, and using a Shirley background. Dimensional analysis (DLS) was performed on a Malvern Panalytical Zetasizer Advance Pro Red (Malvern Panalytical Ltd., Malvern, UK). The AFM imaging was performed using an Ntegra Spectra–NT-MDT instrument (NT-MDT BV, Apeldoorn, The Netherlands) operated in tapping mode. Silicon cantilever tips (NSG 10) with a resonance frequency of 140–390 kHz, a force constant of 5.5–22.5 Nm^−1^, and 10 nm tip curvature radius were used. The HR-SEM micrographs were recorded with a Carl Zeiss NEON 40EsB Cross Beam System (Zeiss Microscopy, Jena, Germany) with thermal Schottky field emission emitter and accelerated Ga ions column. The samples were deposited on the analysis pad from a freshly prepared aqueous and chloroform dispersions of CNDs. The HR-STEM micrographs were recorded with a Thermo Fisher Verios G4 equipped with BF/DF/HAADF-STEM detectors. The EtOH-dispersed CND samples were deposited on the analysis grids and allowed to dry prior to investigation. Freshly prepared samples were investigated through steady-state fluorescence emission spectra and were recorded on a Horiba Fluoromax 4P spectrofluorometer (Horiba Ltd., Kyoto, Japan). The absolute photoluminescence quantum yield (PLQY) values were recorded with the Quanta Fi integration sphere controlled by the Horiba spectrofluorometer using FluorEssence software (ver. 3.5.1.20), according to the equipment manufacturer’s procedure. Excited states’ lifetimes were investigated on the same equipment with the attached Horiba Lifetime module, using a 370 nm LED excitation source.

### 2.3. Preparation of the Argan-Waste-Derived CNDs and Polymer–CND Nanocomposites

Argan cake waste (coarse grinded and dried at 120 °C for 48 h) generated during argan oil preparation through cold pressing was used as precursor. The as-received argan cake waste pellets were ground to a fine powder with an average 100–150 μm size, and further dried prior to thermal processing.

**Figure 1:**
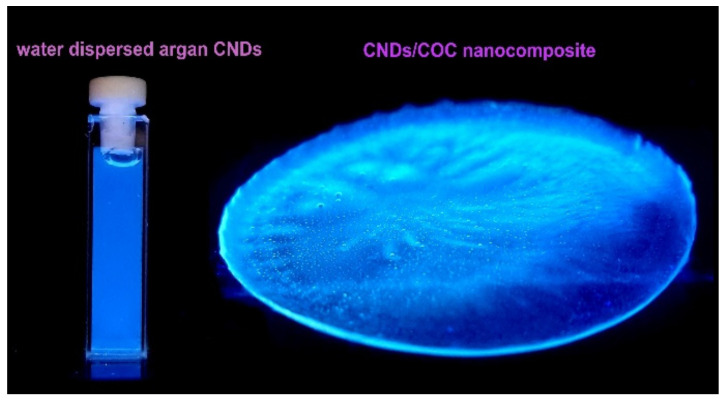
Prepared argan-derived-CNDs dispersed in water and a thick film of CND/COC nanocomposite under UV-A excitation.

For the preparation of the CNDs, a partial carbonization of the argan waste powders was performed under normal atmospheric conditions. In a typical experimental procedure, ca. 0.5 g of argan waste powders are introduced in an open quartz tube reactor provided with a glass heating jacket and exposed at 280 °C for 15 min using a temperature/flow regulated hot air gun (the temperature is reached in ca. 5 s) (Figure S1, Supplementary Information). After the 15 min, the remaining mass in quartz tube is suddenly flooded with cold (3–4 °C) water or cold THF/PSA solutions (0.8 g PSA in 25 mL THF), CLF/COC (0.5 g COC in 20 mL CLF; COC is dissolved under reflux at 62–63 °C and vigorous stirring for at least 1–2 h, given its high resistance to solvents). Approximately 10 mL of the solvents (water/CLF/THF or PSA/THF, COC/CLF) are used to flood the hot carbonized mass resulted after thermal exposure. In our previous works [11,21,43], it was found that the rapid flooding of the hot carbonized mass is critically important in order to obtain CNDs with intense PL emission, most probably due to the sudden cooling of the hot reaction mass when a favorable configuration of the carbonaceous core is achieved. The resulting dispersions are centrifuged at 15,000 rpm for about 15 min. After centrifugation, the final dispersions are optically clear under environmental illumination and present an intense blue PL emission under UV-A/UV-B excitation (Figure 1). The yield of CNDs obtained in the synthesis (evaluated upon evaporation of the solvent in an oven) was ca. 0.1 g CNDs. The COC–CND and PSA–CND thin films were obtained by using a commercially available airbrush with a 0.2 mm nozzle supplied with compressed air. The spray coating operations were performed in a dust free environment. The samples were deposited according to their destination on test PV cells or glass and mica slides for further investigation of the quality of the thin films.

## 3. Results and Discussion

As mentioned above, the structural configuration of CNDs has an essential role in their photoluminescence emission properties. Hence, we have performed a thorough morpho-structural characterization of the CNDs from argan wastes, so as to link their physicochemical properties with the observed PL emission, both in aqueous suspensions and embedded in polymer nanocomposites.

### 3.1. Physicochemical Characterization of the Prepared CNDs

The overall composition of the argan cake waste used as a precursor is presented in Table 1, along with that of the obtained CNDs. The wide XPS survey spectra and deconvoluted data of the C1s, O1s, and N1s regions are shown in the Supplementary Information (Figures S2–S4).

It should be noted that there is a rather good agreement between the composition evaluated by EDX and XPS (with the exception of nitrogen for the precursor), indicating a homogeneous composition of the materials. As seen, the thermal treatment of the argan waste resulted in an overall increase in the carbon content from 74.4 to 81.5 wt.% and a marked decrease in the oxygen content (16.8 to 9.1 wt.%), while the nitrogen content remained rather similar. The carbon enrichment of a biomass precursor upon the thermal treatment is expected, due to the removal of volatile matter (mainly oxygen containing groups) during the treatment. Interestingly, the temperature is low enough to preserve most of the nitrogen content. The XPS spectra of the C1s region (Figures S3 and S4) were deconvoluted into various contributions at binding energies of 284.6 eV (Csp^2^), 286.2 eV (C-O bonds), and 286.7 eV (C=O bonds). The large contributions of the peaks at 284.6 eV (78 at.%) and 285.7 eV (8 at.%) for the C1s region confirmed the predominant aromatic structure of the CNDs. The XPS spectra of the O1s region show the presence of quinone and hydroxyl moieties.

**Figure 2:**
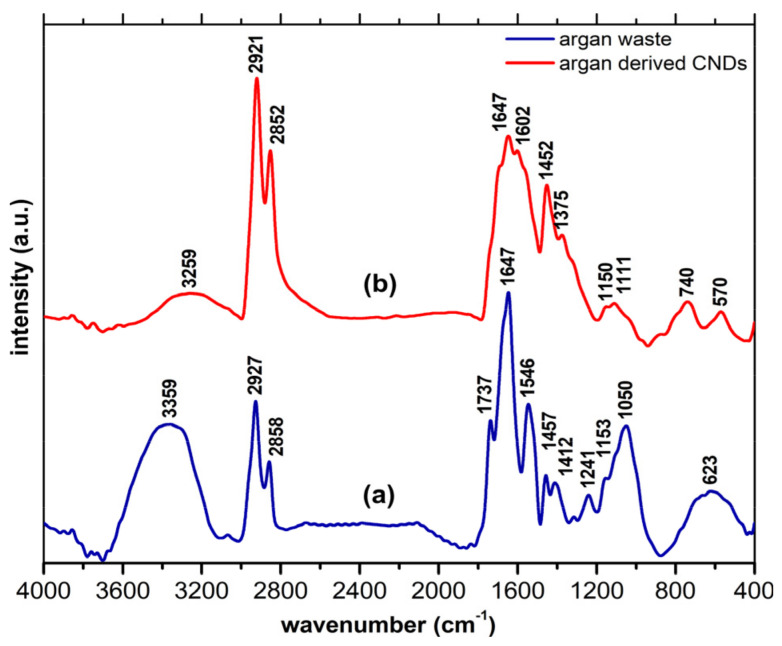
Record FT−IR spectra of the (**a**) argan cake waste and (**b**) derived CNDs.

FTIR analysis was performed for both the argan cake waste precursor and the vacuum-dried CND powders obtained upon thermal processing and cooling in water as a dispersion medium (Figure 2a,b). As seen, the precursor presents a wide signal located between 3359–3259 cm^−1^, related to the bending and stretching vibrations of -OH bonds and attributed to hydroxyl groups and intercalated water [45]. As expected, the contribution of this signal decreased significantly upon the thermal treatment of the precursor to render the CNDs. The bands at 2927/2858 cm^−1^, characteristic of the stretching vibrations of C-H in sp^3^ bonds, were detected in both samples, although they were slightly displaced to lower wavenumbers (2921/2852 cm^−1^) in the CNDs, suggesting some re-arrangements of the carbon within the graphitic core. The presence of various types of fatty acids in the argan waste is highlighted by the bands related to carbonyl groups (1737–1647 cm^−1^). The precursor also displayed a band located at 1546 cm^−1^ associated to N-O stretching. Both former bands are less evident for the CNDs. The 1457 cm^−1^ (-C-H bending) low-intensity peak is slightly down shifted at 1452 cm^−1^ in the case of CNDs. The low-intensity bands located at 1241 and 1153 cm^−1^ are attributed to -C-N and -C-O stretching vibrations, and they are observed in both samples. The band associated with the C=C stretching vibrations of hybridized sp^2^ carbons (~1580–1602 cm^−1^) and the band located at 740 cm^−1^ associated to the -C=C- bending vibrations were only observed for the CNDs; this confirms the aromatization of the carbon matrix during the thermal treatment.

**Figure 3:**
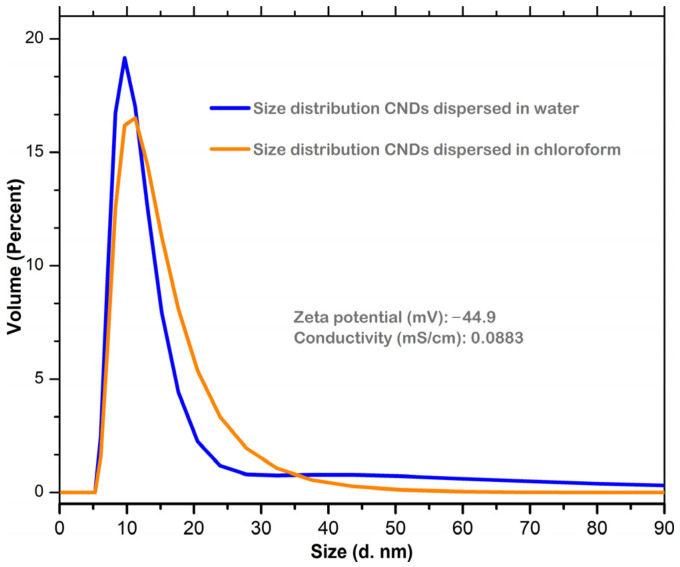
Average hydrodynamic diameter distribution of CND particles dispersed in water and chloroform, obtained by dynamic light scattering.

Dynamic light scattering (DLS) was employed to analyze the particle size of the CNDs dispersed in water and chloroform (Figure 3). As seen, the average hydrodynamic diameter of the CND particles dispersed in water is mostly located within the 6–21 nm range, with the largest fraction (19.17%) at 10 nm. On the other hand, the CND particles dispersed in chloroform displayed a narrower distribution of particle sizes, with the average hydrodynamic diameter in the 6–32 nm range, and the largest fraction (58.3%) within the 9–13 nm range. Furthermore, the zeta potential recorded for the CNDs dispersed in water is −44.9 mV, which indicates a moderate to good stability of the dispersion. The narrower size distribution recorded for chloroform-dispersed CNDs indicates the lower agglomeration tendency of the CNDs in this solvent, compared to water. This is attributed to polar surface moieties present in the CNDs, as inferred by the XPS and FTIR analysis. The average particle size is in rather good agreement with the particle size analysis obtained from SEM/TEM images (see below).

**Figure 4:**
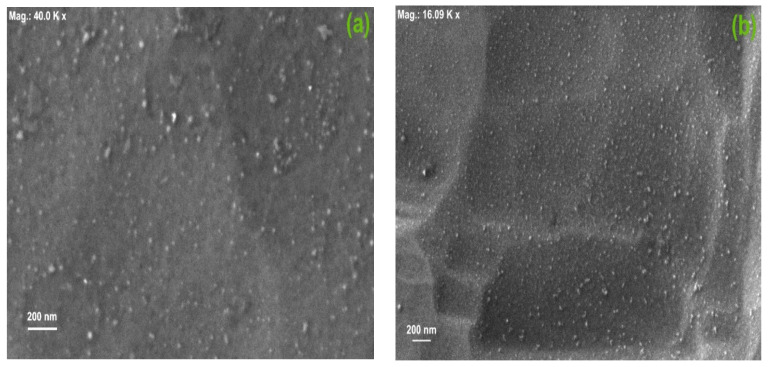
HR-SEM micrographs of the CNDs dispersed in (**a**) water and (**b**) chloroform.

High-resolution SEM micrographs of the CNDs dispersed in water and in chloroform are presented in Figure 4a,b, respectively. Average particle sizes of 25–30 nm were observed for the sample dispersed in water, with some higher-dimension clusters (70–80 nm) clearly visible. This wider distribution of particle sizes is due to the agglomeration tendency in water. For the CNDs dispersed in chloroform, the particle size distribution is markedly lower, with an average size within 15–25 nm and less observation of bigger clusters.

**Figure 5:**
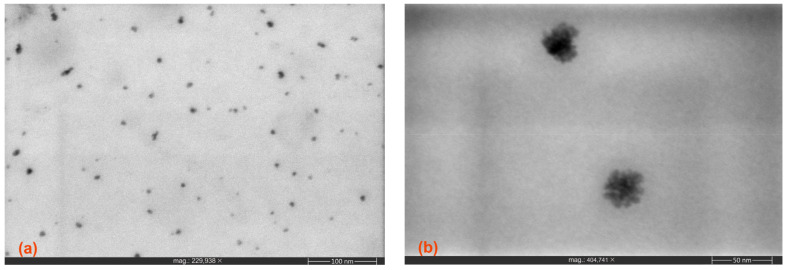
TEM micrographs of the CNDs recorded at (**a**) 230 k and (**b**) 405 k magnifications.

Figure 5a,b presents the TEM micrographs of the prepared CNDs. The samples were prepared by dispersing the CNDs in ethanol, and further allowed to dry on the grids. As seen, nanostructures with dimensions within the 5–20 nm range and a reasonable narrow size distribution were obtained (Figure 5a). Figure 5b provides an interesting close up view of singular nanostructures, which clearly sustains the cluster organization of much smaller individual entities (approx. 5 nm).

### 3.2. Characterization of the CND/Polymer Nanocomposites

**Figure 6:**
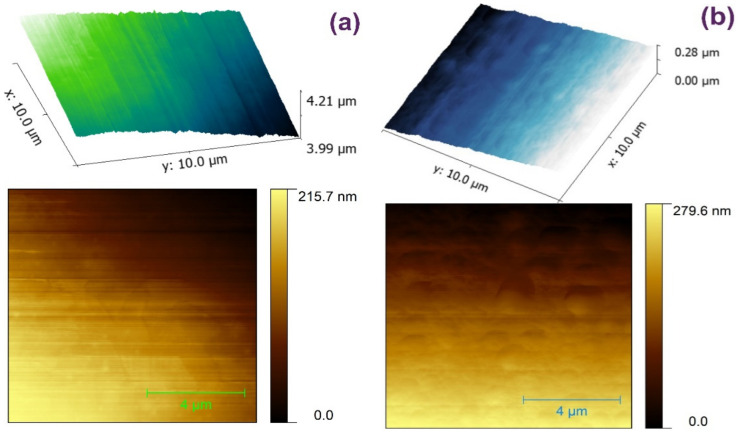
AFM images of the CND/polymer nanocomposites: (**a**) CND/COC and (**b**) CND/PSA.

Figure 6a,b displays the AFM images recorded for the prepared CND/polymer nanocomposites: CND/PSA and CND/COC. The films were deposited on mica through spray coating. The films prepared from CND/COC (Figure 6a) are smooth and homogenous in appearance, with a relatively low density of height irregularities (these are most likely due to small variations of compressed air pressure during the spray coating). In case of the CND/PSA (Figure 6b), the density of height variations is slightly higher, with several regular patterns observed that affect the smoothness of the surface. We attribute these patterns to the rapid drying of the nanocomposite on the mica slide. Anyhow, these small height variations observed in both nanocomposites do not modify the optical transparency of the deposited layers (see below).

### 3.3. Photoluminescence Features of the CNDs and the CND/Polymer Nanocomposites

**Figure 7:**
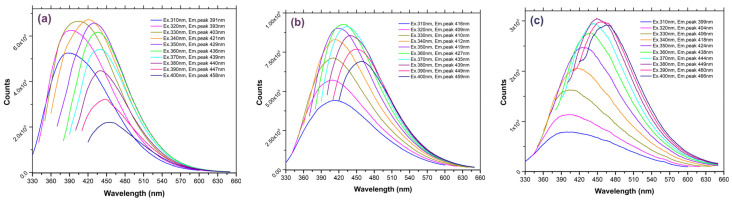
Steady-state PL emission of the argan-prepared CNDs dispersed in (**a**) water, (**b**) chloroform, and (**c**) tetrahydrofuran.

The photoluminescence (PL) features of the prepared carbon nanostructures were investigated both in the suspensions (water and chloroform) and after immobilization in the polymers (CND/polymer nanocomposites). Figure 7a–c presents the steady-state emission spectra of the argan-derived CNDs dispersed in water, chloroform (CLF), and tetrahydrofuran (THF), respectively. As indicated above, the solvents were chosen based on their ability to dissolve the polymers (see experimental section). All the emission spectra were recorded within a 310–400 nm excitation range (10 nm step).

As seen, wide-range emission bands were obtained in all the solvents (average FWHM 50 nm). For the CNDs dispersed in water, the emission bands are centered between 391–458 nm, with the most intense emission at 421 nm under 340 nm excitation. For the CNDs dispersed in CLF, the emission bands were located in the 416–459 nm range, while the most intense emission signal is located at 427 nm under 360 nm excitation. Finally, for the CNDs dispersed in THF, the emission bands are located between the 399–466 nm range and the most intense emission signal at 449 nm was achieved under 380 nm excitation.

For the water-dispersed CNDs, the Stokes shift (i.e., difference between the spectral position of the maximum of the absorption band and the maximum of the emission band) is slightly higher (ca. 79 nm at 350 nm excitation) than that of the CNDs dispersed in CLF (ca. 69 nm at 350 nm excitation) and the CNDs dispersed in THF (ca. 74 nm at 350 nm excitation). For the CNDs dispersed in water, the emission of low-energy photon results are attributed to the presence of -OH moieties in the vicinity of the emissive sites [46]. This is supported by the XPS analysis.

**Figure 8:**
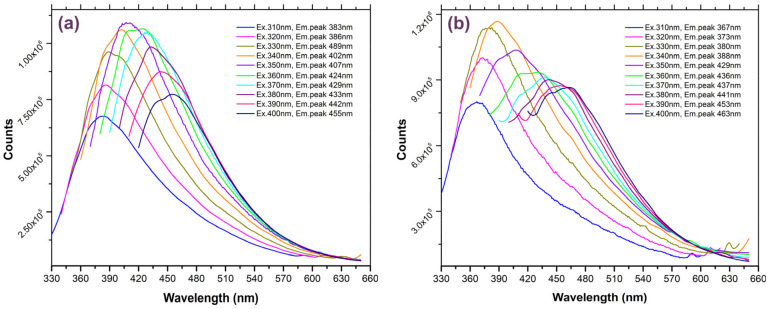
Steady-state PL emission of the (**a**) COC/CND and (**b**) PSA/CND nanocomposites.

Figure 8a,b displays the emission spectra recorded for the CNDs immobilized in the different polymers: COC/CND and PSA/CND nanocomposites, recorded within the same 310–400 nm excitation range. It should be noted that both nanocomposites presented an intense PL emission (ca. 2 times more intense than that of the CNDs dispersed in water and 3–4 times compared with CND–CLF/THF dispersions). According to our tests, the PL emission remained unchanged and stable for several months (tested over four months).

As shown in Figure 1, upon excitation with a regular UV-A source, the dominant emission of both nanocomposites is located in the blue area of the visible spectrum. In particular, the COC/CND nanocomposite PL emission band ranged between 383 and 455 nm, the most intense contribution being located at 407 nm under 350 nm excitation (Figure 8a). In contrast, the emission of PSA/CND is located within the 367–463 nm range, with the most intense emission contribution at 388 nm under a 340 nm excitation wavelength (Figure 8b). The most notable difference compared to the emission features of the CNDs dispersed in the different solvents is the markedly smaller Stokes shift of the immobilized CNDs, particularly for COC/CND (ca. 57 nm for COC/CND, and 79 nm for PSA/CND, under 350 nm excitation). Besides the improved PL emission stability, the rigid environment provided by the polymer backbone seems to also improve the efficiency of the radiative processes. This is inferred from the absolute PLQY yield values recorded for the immobilized and non-immobilized CNDs (Table 2).

For the dispersed CNDs, the highest PLQY values were recorded at a 350 nm excitation wavelength and followed the trend of water > CLF > THF, with a value when using water as the dispersion medium (ca. 15.1%) of almost twice and three times higher than those obtained upon dispersion in CLF and THF, respectively. Interestingly, this trend does not follow a clear correlation with the polarity of the solvents (e.g., dielectric constants of 80 for water; 7.4 for THF; and 4.7 for CLF), suggesting the important role of the surface charges. For the CND/polymer nanocomposites, the PLQY followed the trend of CND/COC (29.6%) > CND/PSA (18%), even though both values are higher than those of the dispersed carbon nanostructures. In this case, the maximum PLQY was obtained upon excitation at 310 nm. The values upon excitation at 350 nm were still higher or comparable to those of the dispersed CNDs. This confirms the improved efficiency of the radiative process upon the embedment in the polymer matrix.

**Figure 9:**
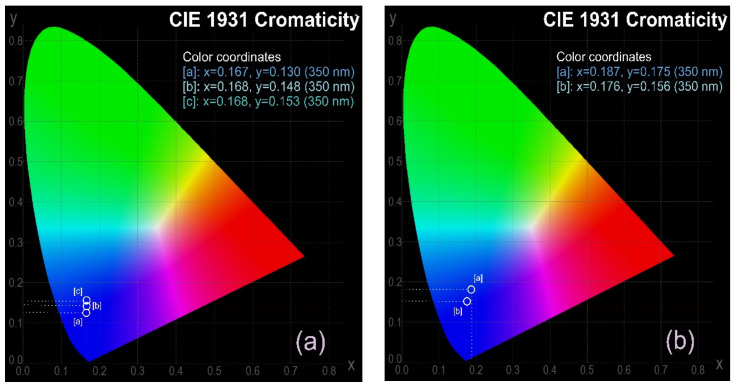
PL emission chromaticity parameters (CIE1931) of the (**a**) solvent-dispersed CNDs and the (**b**) CND/polymer nanocomposites.

Figure 9a,b shows a comparison of the chromatic parameters (according to the CIE 1931 chromaticity co-ordinates) between the solvent-dispersed CNDs and those of the CND/polymer nanocomposites recorded under 350 nm excitation. As seen, the chromaticity co-ordinates of the immobilized CNDs were slightly shifted to higher luminance (brightness, y co-ordinate) and chromaticity (color, x co-ordinate), thus demonstrating the improved stability of the PL features of the former.

**Figure 10:**
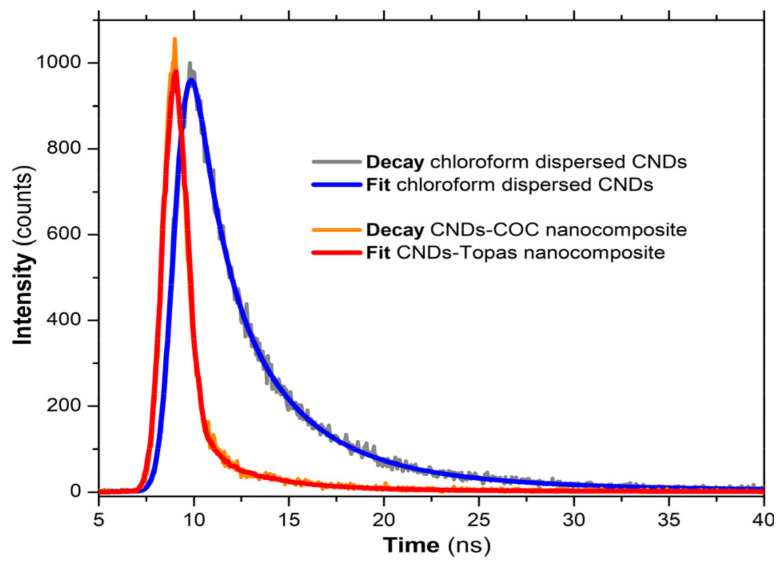
PL lifetime decay recorded for the CLF-dispersed CNDs and COC–CND nanocomposites.

The excited states’ lifetime recorded for both CLF-dispersed CNDs and COC–CND nanocomposites revealed radiative processes within the nanosecond range, as presented in Figure 10 and detailed in Table 3.

Furthermore, the lifetime of the excited states of the CND/polymer nanocomposites was within the nanosecond range (Table 3), typical values for CNDs as reported in the literature [47]. It should be noted that the lifetime of the radiative processes for the CND/polymer nanocomposites was lower than that of the dispersed nanostructures (ca. predominant contribution at 1.05 ns). This agrees with the higher PLQY values. Through embedment in the polymer matrix, the excitation energy transferred to the emissive sites within the CNDs and the resulted photon emission become more efficient.

## 4. Testing the Prepared CND/Polymer Nanocomposites as Photonic Conversion Layers on PV Cells

As mentioned above, the prepared nanocomposites could be easily implemented as photonic conversion layers in a new approach aiming to increase the conversion efficiency of commercially available PV solar cells by taking advantage of the UV radiation contained in the solar spectrum. Most of the available PV solar cells are less sensitive in the upper spectral region of the solar spectrum, the incident UV photons having a low impact on the overall conversion efficiency. Through conversion of the solar UV photons located in the UV-A/UV-B range into the Vis range where the PV cells are mostly sensitive, the overall conversion efficiency could be improved. Typically, the solar radiation UV content at earth level is ~8% depending on the geographical position, elevation, season, time of the day, and specific atmospheric conditions [48]. By harvesting the solar UV photons, which are rather unused in the conversion processes of the commonly available PV cells, an improvement in the overall efficiency could be achieved. Even a small 4–5% conversion efficiency improvement in the UV region could be significant for energy generation where the output power/surface ratio is very important.

**Figure 11:**
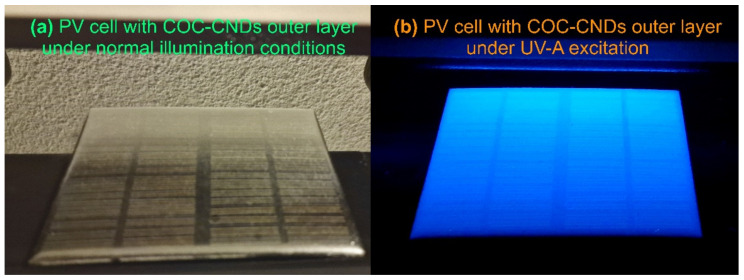
PV cell provided with the COC–CND nanocomposite outer thin layer: (**a**) under normal illumination conditions and (**b**) under UV-A excitation.

In order to test the newly prepared CND/polymer nanocomposites, some commonly available polycrystalline PV cells (model ZW85X115, 12 V, 1.5 W) were provided with an outer photonic conversion layer consisting of the prepared COC–CND nanocomposites. The thin polymeric nanocomposite layer was obtained through spray coating using the methodology and equipment described in Section 2.3. The results are presented in Figure 11a,b.

As could be noted, the deposited COC–CND nanocomposite thin layer present a strong PL emission under UV-A excitation (provided by a 6 W laboratory 365 nm UV lamp) in the blue region of the visible spectrum, thus down-converting the UV photons in a region where the PV cells are more sensitive. Details of the experimental testing layout and measurement setup are presented in Figure S5 (Supplemental Information).

The measurements were performed using a custom-made UV source consisting of an UV LED array powered by an I/V regulated power supply. In order to minimize the measurements errors due to the inherent production batch variance of the PV cells, the same PV cells were measured prior to the application of the COC–CND thin layer with the exactly the same power supply parameters (V/I) of the UV LED array. In each case, the intensity of the LED array UV emission was fine-tuned with a UVAB light meter (RS Pro IM-213) to a value of 5 mW/cm^2^ (roughly corresponding to a 5% UV content of the solar radiation). The output of the tested PV cells was measured without a load resistor using a Tenma 72-7732 data-logging multimeter. The results clearly indicate the conversion efficiency improvement provided by the thin COC–CND photonic conversion layer. Thus, prior to the addition of the outer photonic conversion layer, the PV cell output measured 10.62 V, while the same PV cell provided with the COC–CND conversion layer achieved 11.14 V.

## 5. Conclusions

Blue-emitting polymer/carbon nanodot nanocomposites were prepared through embedding carbon nanodots prepared upon thermal processing of argan cake waste in optically transparent polymer matrices. The as-prepared CNDs dispersed in various solvents presented characteristic excitation wavelength-dependent emission features, with the maximum of the emission located in the blue region of the visible spectrum. The immobilization of the CNDs in optically transparent matrices (e.g., poly(styrene-co-acrylonitrile and cyclo-olefin copolymer) preserved the long-term (blue) emission properties of the CNDs, and improved the quantum efficiency of the radiative processes by twice and three times (achieving a 29.6% PLQY).

The facile processability of the CNDs in thin films of optically transparent polymers is a promising alternative for the implementation of these carbon nanostructures as photonic down-conversion layers in optoelectronics devices. Our preliminary tests of their implementation on commercially available PV cells revealed a notable improvement in the conversion efficiency in the UV range. Current studies are ongoing, aiming to further improve the conversion efficiency of conventional photovoltaic solar cells with these conversion layers by taking advantage of the UV radiation contained in the solar spectrum.

## Figures and Tables

**Table 1:** Elemental composition of the argan waste and the prepared CNDs.

EDX					XPS			
Element	Argan Cake Waste		CNDs		Argan Cake Waste		CNDs	
	wt.%	at.%	wt.%	at.%	wt.%	at.%	wt.%	at.%
Carbon	74.4	78.7	81.5	84.6	78.7	82.9	83.8	86.5
Nitrogen	8.8	8	9.4	8.3	2.6	2.3	7.2	8
Oxygen	16.8	13.3	9.1	7.1	18.7	14.8	9	5.5

**Table 2:** Absolute PLQY values at different excitation wavelengths of the solvent dispersed CNDs and the CND/polymer nanocomposites (maximum values are highlighted in bold).

Excitation (nm)	310	320	330	340	350	360	370	380	390	**400**
CNDs dispersed in H_2_O	CIE 1931 parameter X= 0.167, Y= 0.130 @350 nm									
Absolute PLQY (%)	2.6	12.3	14	13.8	**15.1**	11.3	9.3	7.5	5.3	3.4
CNDs dispersed in THF	CIE 1931 parameter X= 0.168, Y= 0.153 @350 nm									
Absolute PLQY (%)	1.4	3.4	4.1	5.5	**5.9**	5.5	5.2	5	4.9	4.7
CNDs dispersed in CLF	CIE 1931 parameter X = 0.168, Y = 0.148 @350 nm									
Absolute PLQY (%)	8.1	8.1	8.5	8.8	**8.9**	8.7	8.2	8	7.5	6.9
CND/PSA nanocomposite	CIE 1931 parameter X = 0.187, Y = 0.175 @350 nm									
Absolute PLQY (%)	**18**	12.4	12.6	12.1	12	10	8	7.7	8.6	10.1
CND/COC nanocomposite	CIE 1931 parameter X = 0.176, Y = 0.157 @350 nm									
Absolute PLQY (%)	**29.6**	22.4	21.7	23.9	21.8	17.1	14.9	14.9	16.6	18.3

**Table 3:** Recorded excited states’ lifetimes.

CLF Dispersed CNDs		COC–CND Nanocomposites	
Lifetimes (ns)	Contribution (%)	Lifetimes (ns)	Contribution (%)
τ1 = 1.01	20	τ1 = 1.05	84
τ2 = 3.38	55	τ1 = 3.03	12
τ3 = 9.18	25	τ1 = 10.60	4

## Data Availability

The data presented in this study are available upon request from the corresponding author.

## Supplementary Materials

The following supporting information can be downloaded at: https://www.mdpi.com/article/10.3390/nano14010083/s1, Figure S1: Laboratory experimental setup used for CNDs preparation; Figure S2: XPS survey spectra recorded for (a) argan waste cake and (b) prepared CNDs; Figure S3: Deconvolution of the (a) C1s, (b) O1s and (c) N1s regions in the XPS spectra recorded for argan waste cake; Figure S4: Deconvolution of the (a) C1s, (b) O1s and (c) N1s regions in the XPS spectra recorded the prepared CNDs; Figure S5: Experimental testing layout and measurement setup for testing the PV cells provided with the prepared nanocomposites as photonic conversion layers.

## References

[1] Humaera N.A., Fahri A.N., Armynah B., Tahir D. (2021). Natural source of carbon dots from part of a plant and its applications: A review. Luminescence.

[2] Rodríguez-Varillas S., Fontanil T., Obaya Á.J., Fernández-González A., Murru C., Badía-Laíño R. (2022). Biocompatibility and Antioxidant Capabilities of Carbon Dots Obtained from Tomato (*Solanum lycopersicum*). Appl. Sci..

[3] Stepanidenko E.A., Ushakova E.V., Fedorov A.V., Rogach A.L. (2021). Applications of Carbon Dots in Optoelectronics. Nanomaterials.

[4] Yuan T., Meng T., He P., Shi Y., Li Y., Li X., Fan L., Yang S. (2019). Carbon quantum dots: An emerging material for optoelectronic applications. J. Mater. Chem. C.

[5] Barrientos K., Arango J.P., Moncada M.S., Placido J., Patiño J., Macías S.L., Maldonado C., Torijano S., Bustamante S., Londoño M.E. (2023). Carbon dot-based biosensors for the detection of communicable and non -communicable diseases. Talanta.

[6] Das S., Ngashangva L., Goswami P. (2021). Carbon Dots: An Emerging Smart Material for Analytical Applications. Micromachines.

[7] Bhattacharya T., Shin G.H., Kim J.T. (2023). Carbon Dots: Opportunities and Challenges in Cancer Therapy. Pharmaceutics.

[8] Geng B., Hu J., Li Y., Feng S., Pan D., Feng L., Shen L. (2022). Near-infrared phosphorescent carbon dots for sonodynamic precision tumor therapy. Nat. Commun..

[9] Naik K., Chaudhary S., Ye L., Parmar A.S. (2022). A Strategic Review on Carbon Quantum Dots for Cancer-Diagnostics and Treatment. Front. Bioeng. Biotechnol..

[10] Latha B.D., Soumya K., More N., Choppadandi M., Guduru A.T., Singh G., Kapusetti G. (2023). Fluorescent carbon quantum dots for effective tumor diagnosis: A comprehensive review. Adv. Biomed. Eng..

[11] Tiron A., Stan C.S., Luta G., Uritu C.M., Vacarean-Trandafir I.-C., Stanciu G.D., Coroaba A., Tiron C.E. (2021). Manganese-Doped N-Hydroxyphthalimide-Derived Carbon Dots—Theranostics Applications in Experimental Breast Cancer Models. Pharmaceutics.

[12] Liu J., Li R., Yang B. (2020). Carbon Dots: A New Type of Carbon-Based Nanomaterial with Wide Applications. ACS Cent. Sci..

[13] Yadav P.K., Chandra S., Kumar V., Kumar D., Hasan S.H. (2023). Carbon Quantum Dots: Synthesis, Structure, Properties, and Catalytic Applications for Organic Synthesis. Catalysts.

[14] Dam B.D., Nie H., Ju B., Marino E., Paulusse J.M.J., Schall P., Li M., Dohnalová K. (2017). Excitation-Dependent Photoluminescence from Single-Carbon Dots. Small.

[15] Singhal P., Vats B.G., Pulhani V. (2022). Origin of solvent and excitation dependent emission in newly synthesized amphiphilic carbon dots. J. Lumin..

[16] Ding H., Li X.H., Chen X.B., Wei J.S., Li X.B., Xiong H.M. (2020). Surface states of carbon dots and their influences on luminescence. J. Appl. Phys..

[17] Wang B., Lu S. (2022). The light of carbon dots: From mechanism to applications. Matter.

[18] Tiwari A., Walia S., Sharma S., Chauhan S., Kumar M., Gadlyd T., Randhawa T.K. (2023). High quantum yield carbon dots and nitrogen-doped carbon dots as fluorescent probes for spectroscopic dopamine detection in human serum. J. Mater. Chem. B.

[19] Yan F., Jiang Y., Sun X., Bai Z., Zhang Y., Zhou X. (2018). Surface modification and chemical functionalization of carbon dots: A review. Microchim. Acta.

[20] Koç O.K., Üzer A., Apak R. (2022). High Quantum Yield Nitrogen-Doped Carbon Quantum Dot-Based Fluorescent Probes for Selective Sensing of 2,4,6-Trinitrotoluene. ACS Appl. Nano Mater..

[21] Stan C.S., Coroabă A., Ursu E.L., Secula M.S., Simionescu B.C. (2019). Fe(III) doped carbon nanodots with intense green photoluminescence and dispersion medium dependent emission. Sci. Rep..

[22] Singh A., Qu Z., Sharma A., Singh M., Tse B., Ostrikov K., Popat A., Sonar P., Kumeria T. (2023). Ultra-bright green carbon dots with excitation-independent fluorescence for bioimaging. J. Nanostruct. Chem..

[23] Li P., Xue S., Sun L., Zong X., An L., Qu D., Wang X., Sun Z. (2022). Formation and fluorescent mechanism of red emissive carbon dots from o-phenylenediamine and catechol system. Light Sci. Appl..

[24] Ding H., Zhou X.X., Wei J.S., Li X.B., Qin B.T., Chen X.B., Xiong H.M. (2020). Carbon dots with red/near-infrared emissions and their intrinsic merits for biomedical applications. Carbon.

[25] Stan C.S., Gospei Horlescu P., Ursu L.E., Popa M., Albu C. (2017). Facile preparation of highly luminescent composites by polymer embedding of carbon dots derived from N-hydroxyphthalimide. J. Mater. Sci..

[26] Cui L., Ren X., Sun M., Liu H., Xia L. (2021). Carbon Dots: Synthesis, Properties and Applications. Nanomaterials.

[27] Nammahachak N., Aup-Ngoen K.K., Asanithi P., Horpratum M., Chuangchote S., Ratanaphan S., Surareungchai W. (2022). Hydrothermal synthesis of carbon quantum dots with size tunability via heterogeneous nucleation. RSC Adv..

[28] Luo H., Lari L., Kim H., Hérou S., Tanase L.C., Lazarovc V.K., Titirici M.M. (2022). Structural evolution of carbon dots during low temperature pyrolysis. Nanoscale.

[29] Khayal A., Dawane V., Amin M.A., Tirth V., Yadav V.K., Algahtani A., Khan S.H., Islam S., Yadav K.K., Jeon B.H. (2021). Advances in the Methods for the Synthesis of Carbon Dots and Their Emerging Applications. Polymers.

[30] Adejumo I.O., Adebiyi O.A., Saleh H.M. (2021). Agricultural Solid Wastes: Causes, Effects, and Effective Management. Strategies of Sustainable Solid Waste Management.

[31] Raza M.H., Abid M., Faisal M., Yan T., Akhtar S., Adnan K.M.M. (2022). Environmental and Health Impacts of Crop Residue Burning: Scope of Sustainable Crop Residue Management Practices. Int. J. Environ. Res. Public Health.

[32] Kang C., Huang Y., Yang H., Yan X.F., Chen Z.P. (2020). A Review of Carbon Dots Produced from Biomass Wastes. Nanomaterials.

[33] Gedda G., Sankaranarayanan S.A., Putta C.L., Gudimella K.K., Rengan A.K., Girma W.M. (2023). Green synthesis of multi-functional carbon dots from medicinal plant leaves for antimicrobial, antioxidant, and bioimaging applications. Sci. Rep..

[34] Newman Monday Y., Abdullah J., Yusof N.A., Abdul Rashid S., Shueb R.H. (2021). Facile Hydrothermal and Solvothermal Synthesis and Characterization of Nitrogen-Doped Carbon Dots from Palm Kernel Shell Precursor. Appl. Sci..

[35] Xiaoyun Q., Cuicui F., Jin Z., Wenlong S., Xiaomei Q., Yanghai G., Lan W., Huishi G., Fenghua C., Liying J. (2022). Direct preparation of solid carbon dots by pyrolysis of collagen waste and their applications in fluorescent sensing and imaging. Front. Chem..

[36] Zhang J., Xia A., Chen H., Nizami A.S., Huang Y., Zhu X., Zhu X., Liao Q. (2022). Biobased carbon dots production via hydrothermal conversion of microalgae Chlorella pyrenoidosa. Sci. Total Environ..

[37] Prasannan A., Imae T. (2013). One-Pot Synthesis of Fluorescent Carbon Dots from Orange Waste Peels. Ind. Eng. Chem. Res..

[38] González-González R.B., González L.T., Madou M., Leyva-Porras C., Martinez-Chapa S.O., Mendoza A. (2022). Synthesis, Purification, and Characterization of Carbon Dots from Non-Activated and Activated Pyrolytic Carbon Black. Nanomaterials.

[39] Ganguly S., Das P., Banerjee S., Das N.C. (2019). Advancement in science and technology of carbon dot-polymer hybrid composites: A review. Funct. Compos. Struct..

[40] Maxim A.A., Sadyk S.N., Aidarkhanov D., Surya C., Ng A., Hwang Y.-H., Atabaev T.S., Jumabekov A.N. (2020). PMMA Thin Film with Embedded Carbon Quantum Dots for Post-Fabrication Improvement of Light Harvesting in Perovskite Solar Cells. Nanomaterials.

[41] Liu Y., Wang P., Shiral Fernando K.A., LeCroy G.E., Maimaiti H., Harruff-Miller B.A., Lewis W.K., Bunker C.E., Hou Z.L., Sun P.Y. (2016). Enhanced fluorescence properties of carbon dots in polymer films. J. Mater. Chem. C.

[42] Bhunia S.K., Nandi S., Shiklerb R., Jelinek R. (2016). Tuneable light-emitting carbon-dot/polymer flexible films prepared through one-pot synthesis. Nanoscale.

[43] Shebeeb C.M., Joseph A., Chalikkara F., Dinesh R., Sajith V. (2022). Fluorescent carbon dot embedded polystyrene particle: An alternative to fluorescently tagged polystyrene for fate of microplastic studies: A preliminary investigation. Appl. Nanosci..

[44] Gharby S., Charrouf Z. (2022). Argan Oil: Chemical Composition, Extraction Process, and Quality Control. Front. Nutr..

[45] Zhang C., Dabbs D.M., Liu L.M., Ilhan A., Aksay I.A., Car R., Selloni A. (2015). Combined Effects of Functional Groups, Lattice Defects, and Edges in the Infrared Spectra of Graphene Oxide. J. Phys. Chem. C.

[46] Kamel B., El-Daher M.S., Bachir W., Ibrahim A., Aljalali S. (2022). Effect of Solvents on the Fluorescent Spectroscopy of BODIPY-520 Derivative. J. Spectrosc..

[47] Barman M.K., Patra A. (2018). Current status and prospects on chemical structure driven photo luminescence behaviour of carbon dots. J. Photochem. Photobio. C.

[48] Chadyšienė R., Girgždiene R., Girgždys A. (2005). Ultraviolet radiation and ground level ozone variation in Lithuania. J. Environ. Eng. Landsc. Manag..

