# Supplementary material for: Photoluminescence of Argan-Waste-Derived Carbon Nanodots Embedded in Polymer Matrices
Source: Nanomaterials (Basel). 2023 Dec 27;14(1):83. doi: 10.3390/nano14010083 (PMC10780386; doi:10.3390/nano14010083)
Supplement: Supplementary file 1 [file nanomaterials-14-00083-s001.zip › nanomaterials-2758350-supplementary.pdf]

*Supplementary Materials*

# Photoluminescence of Argan-Waste-Derived Carbon Nanodots Embedded in Polymer Matrices

**Corneliu S. Stan [1,*], Noumane Elouakassi [2], Cristina Albu [1], Ania O. Conchi [3], Adina Coroaba [4], Laura E. Ursu [4], Marcel Popa [1,5,*], Hamid Kaddami [2,6] and Abdemaji Almaggoussi [2,7]**

[1] Faculty of Chemical Engineering and Environmental Protection, Gh. Asachi Technical University of Iasi, D. Mangeron 73 Ave., 700050 Iasi, Romania; cristina.albu@tuiasi.ro
[2] Innovative Materials for Energy and Sustainable Development (IMED-Lab), Faculty of Science and Technology, Cadi Ayyad University, Av. Abdelkrim Khattabi, B.P. 511, Marrakech 40000, Morocco; noumanelouaskassi0641@gmail.com (N.E.); h.kaddami@uca.ma (H.K.); a.almaggoussi@uca.ma (A.A.)
[3] Conditions Extremes Matériaux Haute Temperature et Irradiation (CEMHTI), UPR 3079, CNRS, Université d'Orléans, 45100 Orleans, France; conchi.ania@cnrs-orleans.fr
[4] Centre of Advanced Research in Bionanoconjugates and Biopolymers, "Petru Poni" Institute of Macromolecular Chemistry, Grigore Ghica Voda 41A Alley, 700487 Iasi, Romania; adina.coroaba@icmpp.ro (A.C.); ursu.laura@icmpp.ro (L.E.U.)
[5] Academy of Romanian Scientists, Ilfov Street, 050054 Bucharest, Romania
[6] Sustainable Materials Research Center (SusMat-RC), Lot 660-Hay Moulay Rachid, Ben Guerir 43150, Morocco
[7] Applied Chemistry and Engineering Research Centre of Excellence (ACER CoE), Advanced Organic Optoelectronic Laboratory, Mohammed VI Polytechnic University (UM6P), Lot 660-Hay Moulay Rachid, Ben Guerir 43150, Morocco
* Correspondence: stancs@tuiasi.ro (C.S.S.); marpopa@ch.tuiasi.ro (M.P.)

# Electronic Supporting Information

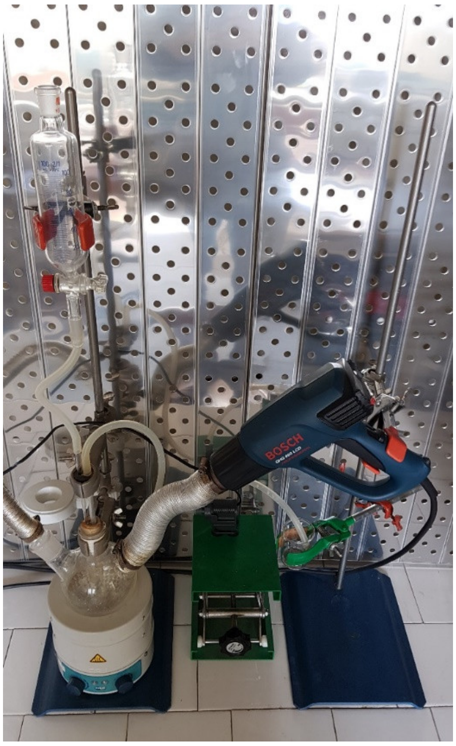

**Figure S1**. Laboratory experimental setup used for CNDs preparation.

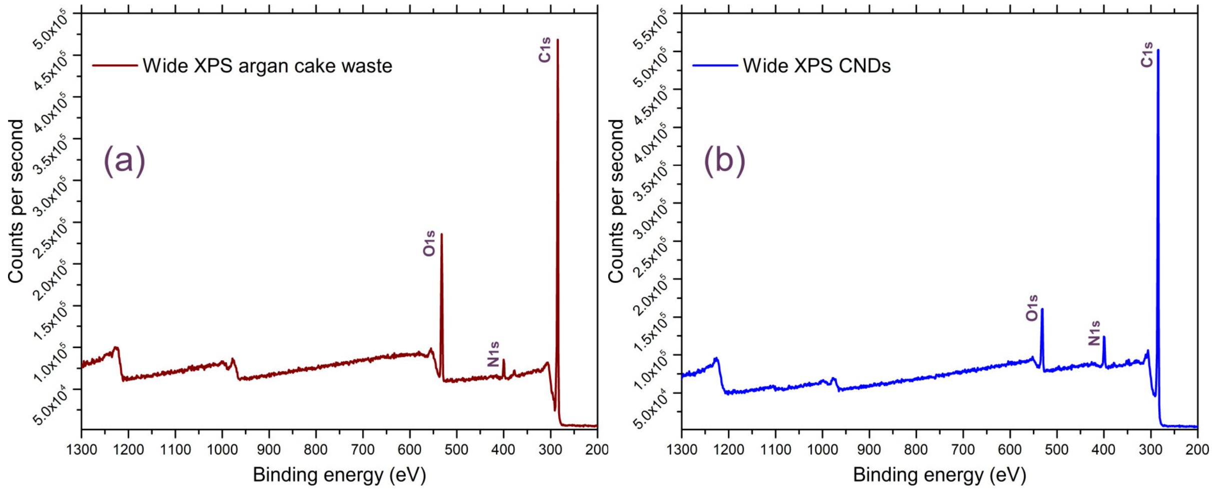


**Figure S2.** XPS survey spectra recorded for (**a**) argan waste cake and (**b**) prepared CNDs.

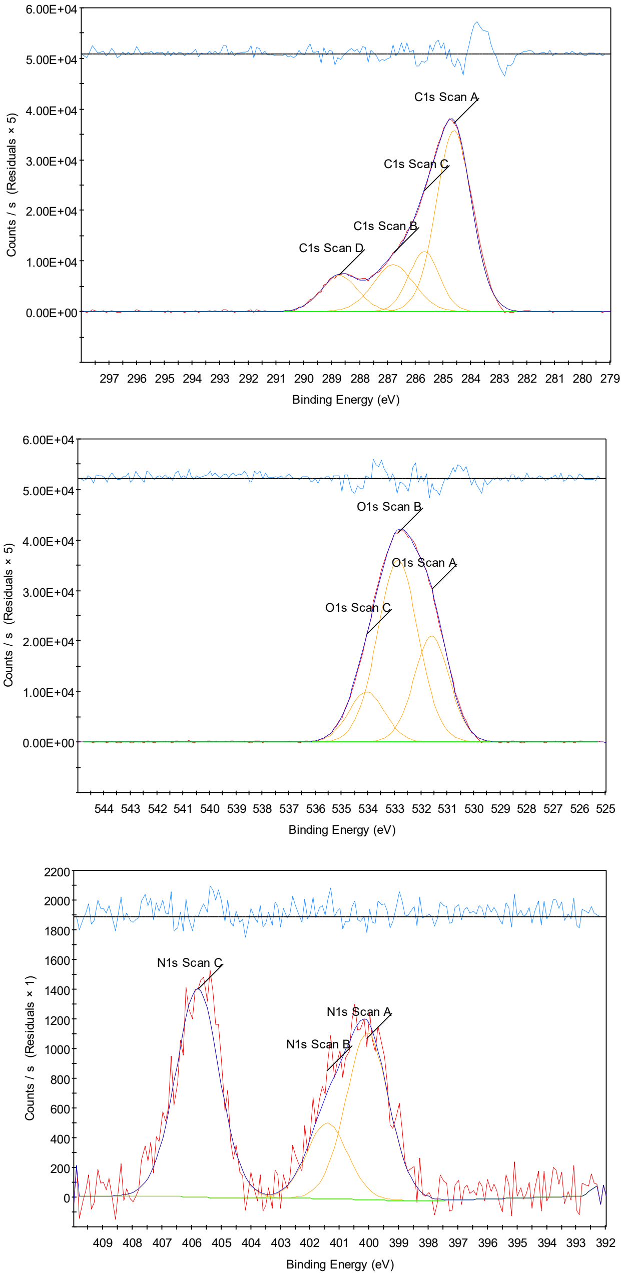


**Figure S3**. Deconvolution of the (**a**) C1s, (**b**) O1s and (**c**) N1s regions in the XPS spectra recorded for argan waste cake.

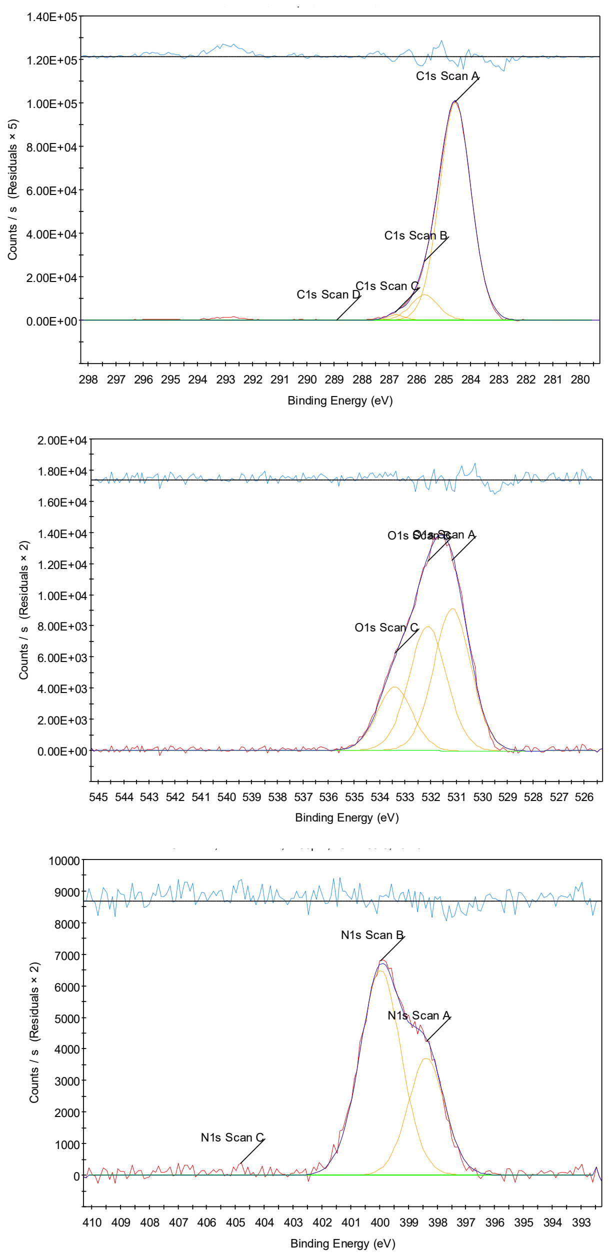


**Figure S4**. Deconvolution of the (a) C1s, (b) O1s and (c) N1s regions in the XPS spectra recorded the prepared CNDs.

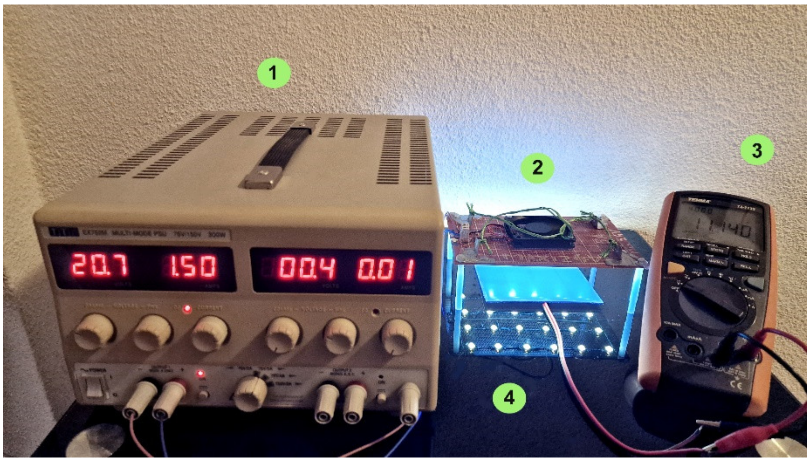


**Figure S5**. Experimental testing layout and measurement setup for testing the PV cells provided with the prepared nanocomposites as photonic conversion layers.

1. TTI EX752M I/V regulated multi-mode power supply
2. UV-LED array (24x UVLED 140°; 1400mA; P: 6300mW; 360÷370nm; 3,5÷4,5VDC - Optosupply OSV1XDE5E1E
3. Tenma 72-7732 data-logging multimeter
4. test PV cell (model ZW85X115, 12V, 1.5W) provided with the prepared COC-CNDs nanocomposite as photonic conversion layer
